# SAA1 Protein: A Potential Biomarker for Acute Myeloid Leukemia

**DOI:** 10.3390/biomedicines13040880

**Published:** 2025-04-05

**Authors:** Pedro Leite Azevedo, Mayara Rezende, Milena Felix, Stephany Corrêa, Eliana Abdelhay, Renata Binato

**Affiliations:** Stem Cell Laboratory, National Cancer Institute (INCA), Rio de Janeiro 20230-130, RJ, Brazil; rezendema@hotmail.com (M.R.); felixxmilenaa@gmail.com (M.F.); stephany.correa@inca.gov.br (S.C.); eabdelhay@inca.gov.br (E.A.); renata.binato@inca.gov.br (R.B.)

**Keywords:** acute myeloid leukemia (AML), proteomic, diagnostic biomarker

## Abstract

**Background/Objectives:** Despite its heterogeneity and diagnostic challenges, acute myeloid leukemia (AML) originates from stem cell transformation and alterations in the hematopoietic niche (HN) could be related to leukemic transformation. Therefore, the aim of this study was to evaluate the protein profile of HN from AML patients and compare it with the profile of healthy donors (HDs). **Methods:** A proteomic analysis was conducted to identify differentially expressed (DE) proteins in BM plasma from AML patients and HD. In silico analysis was performed to identify biological processes and signaling pathways involved. Additionally, ELISA confirmed the expression of the DE protein of interest in BM plasma samples. **Results:** Proteomic analysis revealed alterations in the plasma profiles of AML patients and 36 DE proteins were found. Among then, we highlight C8G, CFB, SAA1, SERPINA3 and SERPINC1, which are related to inflammatory response process. Thus, considering the role of the secreted protein SAA1 in the inflammatory context and that it is described as a potential biomarker in several tumors, we selected SAA1 for ELISA confirmation. The results corroborated our findings, indicating that increased expression of SAA1 could be related to AML. Our results also revealed that SAA1 can stimulate immune signaling through NF-kappa-B activation. **Conclusions:** These findings position SAA1 as a promising biomarker for AML diagnosis, offering a potential tool for more accurate identification of the disease. Nevertheless, further studies are needed to understand the relationship of SAA1 with the leukemic transformation process in AML and its potential clinical use.

## 1. Introduction

Acute myeloid leukemia (AML) is an aggressive clonal myeloid neoplasm characterized by maturation arrest of myelopoiesis, leading to the accumulation of myeloblasts in the bone marrow (BM) and/or peripheral blood [1]. The global incidence of AML has been increasing over time. In 2021, approximately 144,645 individuals were diagnosed worldwide, with a higher prevalence in men than in women. The disease affects primarily adults, and its incidence rises with age; it can also affect a small percentage (10–15%) of children. Despite advancements in treatment, the prognosis for AML remains poor, particularly among older patients. The five-year survival rate for all adult AML patients is below 50%, and although 40–50% of elderly patients may achieve complete remission, the five-year survival rate in this population drops to just 5% due to the high rate of relapse [2].

Timely diagnosis and effective treatment of AML are crucial for managing the disease [3]. The World Health Organization (WHO) system categorizes AML on the basis of clinical, morphological, immunophenotyping, cytogenetic and molecular genetic characteristics at initial diagnosis [4,5]. In addition, AML can still be classified by the European Leukemia Net (ELN), which classifies AML on the basis of genetic abnormalities and defines the disease into risk categories [6]. Despite significant progress in understanding its pathogenesis and treatment modalities, AML diagnosis remains a clinical challenge because of its diverse molecular landscape and complex genetic alterations [7].

Although AML is a heterogeneous disease and patients present different characteristics at diagnosis, in 1994, it was postulated that AML has a unique origin on the basis of the malignant transformation of hematopoietic stem cells (HSCs) into leukemic stem cells (LSCs) [8]. In this context, the bone marrow microenvironment, also called the hematopoietic niche (HN), could play a role in this leukemic transformation [9].

A healthy HN is composed of different components and promotes coordinated signaling to regulate HSC functions and hematopoiesis processes [10]. The extracellular matrix, proteins and metabolites present in the HN are essential for regulating the differentiation and maintenance of HSCs in the BM, and changes in this signaling pathway have already been associated with hematopoietic insufficiency. Thus, HN is a potential factor responsible for promoting changes in HSCs, and these changes could culminate in AML [11].

Given that AML has a unique origin and that the HN may be capable of altering HSCs, it would be interesting to evaluate whether the HN of patients with AML is altered. For this purpose, high-throughput proteomic techniques, particularly mass spectrometry, are used to discover clinically relevant biomarkers in AML patients [12,13,14,15].

At present, the most used diagnostic biomarkers in use for AML include the identification of mutations in the *FLT3*, *NPM1* and *CEBPA* genes and cytogenetic abnormalities such as t(8;21), inv(16) and t(15;17). However, not all AML patients exhibit these alterations, adding to the complexity of diagnosis. Importantly, no bone marrow plasma biomarker is currently established for use at the time of AML diagnosis in clinical practice. Therefore, the identification of new biomarkers for this complex disease, through simplified methodologies such as ELISA, may contribute to a more accurate and earlier diagnosis, in addition to assisting in risk stratification and the monitoring of response to treatment [6].

In this context, the aim of this study was to evaluate the HN protein profile of AML patients and compare it with the HN protein profile of healthy donors through proteomic analysis of BM plasma to identify differentially expressed proteins that could be potential biomarkers for AML regardless of the categorization and risk classification of AML. After proteomic analysis, we identified 36 commonly differentially expressed proteins in BM plasma from all AML patients in comparison with BM plasma from healthy donors. Among these proteins, SAA1 was upregulated in all AML patients. Serum amyloid A1 (SAA1) is an inducible acute phase protein in response to injury, infection and inflammation and has been identified as a novel proinflammatory oncoprotein whose levels progressively increase with AML progression. SAA1 also selectively promotes the growth of patient-derived AML cells independent of cytogenetic or mutational profile [16].

This protein is involved in several altered processes in AML and has been used as a diagnostic biomarker for other types of cancer. Therefore, our results are promising and reinforce that SAA1 can be used as a potential diagnostic biomarker for AML.

## 2. Materials and Methods

### 2.1. Samples from Patients and Healthy Donors

The samples used in this study were from the bone marrow of patients with AML at diagnosis without any treatment and also from healthy donors (HDs) that were registered at the Bone Marrow Transplantation Unit, National Cancer Institute (INCA) (Rio de Janeiro, Brazil) which were used as control (Table 1). To characterize AML samples, we used the French–American–British (FAB) classification system, which is defined by specific morphological characteristics and differentiation stages and classified into different subtypes. In addition to the FAB classification, we characterized AML samples according to ELN risk classification (Table 2) [4,5,6,17]. The samples were stratified into two cohorts: the proteomic cohort and the ELISA cohort. All samples were obtained in accordance with the local Ethics Committee guidelines and the Declaration of Helsinki. The INCA Ethics Committee approved this study (No. 06281419.0.0000.5274), and all participants signed informed consent forms.

### 2.2. Proteomic Study

#### 2.2.1. Bone Marrow Plasma Preparation

BM samples from AML patients and HDs were collected in tubes containing EDTA (ethylenediaminetetraacetic acid) anticoagulant (BD, Franklin Lakes, NJ, USA). Each sample was centrifuged at 1500 rpm for 10 min, after which the plasma was transferred to a cryotube, and 1 μL/mL Mix-Protease Inhibitor (GE Healthcare, Chicago, IL, USA) was added. The samples were frozen at −70 °C for further analysis.

#### 2.2.2. Protein Quantification of Plasma Samples

The quantification of BM plasma proteins was performed according to the Bradford method [18]. Quantification was performed in triplicate for each BM plasma sample. The BM plasma samples were read on a Biophotometer™ (Eppendorf, Hamburgo, Germany) at an absorbance of 595 nm.

#### 2.2.3. In-Solution Tryptic Digestion

After protein quantification, the BM plasma samples used for proteomic analysis were grouped into four pools according to the FAB criteria, concentrated 39× and exchanged with 50 mM NH4HCO3 via a 3 kDa ultrafiltration device (Millipore, Burlington, MA, USA). After that, 200 μg of protein from each pool was denatured (0.1% RapiGEST SF at 60 °C for 15 min) (Waters, Milford, MA, USA), reduced with 10 mM DTT (Dithiothreitol) at 60 °C for 30 min, and alkylated at room temperature in the dark with 10 mM iodoacetamide for 30 min to then be enzymatically digested with trypsin at a 1:50 *w*/*w* enzyme/protein ratio (Promega, Madison, WI, USA). To stop the digestion, 10 μL of 5% TFA (Trifluoroacetic acid) was added, and, after that, yeast alcohol dehydrogenase (ADH; P00330, Waters, Milford, MA, USA) was added to a final concentration of 0.2 pmol/μL, which was consistent with standardized quantification [19].

#### 2.2.4. Label-Free Protein Quantitation by Mass Spectrometry

According to the methods of Pizzatti and colleagues [20], samples were subjected to nanoscale chromatographic separation (2DnanoLC) using the Waters^®^ nanoACQUITY UPLC (ultra-performance liquid chromatography) system [20]. Mass spectrometric analysis was performed as previously described [21]. To ensure that the LC–MS data generated at low energy were effectively acquired at 400 to 2000 *m*/*z* intervals, the mass spectrometry (MS) profile was adjusted. Therefore, we ensured that all *m*/*z* values less than 400 in the LC/MSE were the result of dissociation in the collision cell.

The database search and protein quantification were performed as previously reported [18]. To process the spectra and databank search results, ProteinLynx Global Server v.3.0 (PLGS) with Expression^E^ software was used. The UniProtKB databank (release 2019_06_HUMAN) was used with manually reviewed annotations. For expression analysis using the Expression^E^ tool, only the identified proteins obtained in the Identity^E^ step of PLGS analysis were considered. The proteins identified by the PLGS Expression^E^ tool algorithm were organized into a statistically significant list corresponding to the up- and downregulation ratios between the samples from different groups compared to the control. Additionally, filtering was performed to select only proteins that presented differential expression levels (ratios) with a *p*-value less than 0.05 and fold-change ≥ 2 [17].

#### 2.2.5. In Silico Analysis

Pathway analysis and biological process information related to the differentially expressed (DE) proteins were obtained via KEGG (Kyoto Encyclopedia of Genes and Genomes Release 110.1, 1 May 2024), WebGestalt (WEB-based GEne SeT AnaLysis Toolkit 2024), STRING (Search Tool for the Retrieval of Interacting Genes/Proteins—version 12.0) and the Reactome Pathway Database with Pathway Browser tools (version 89-6/2024). In KEGG, we used a search tool, limiting the selection of signaling pathways to those described in Homo sapiens. In WebGestalt, the method of interest was overrepresentation analysis, with Homo sapiens as the selected organism. For the use of the STRING tool, the selected interaction sources included Text Mining, Experiments, Databases, Coexpression, Neighborhood, Gene Fusion and Co-occurrence, with a minimum required interaction score of 0.4. Networks were evaluated with a maximum of five interactors. Finally, in the Reactome Pathway Database platform with Pathway Browser, searches were performed directly for the protein of interest, allowing the identification of signaling pathways in which these proteins are involved through the “Locations in Pathway Browser” tool.

### 2.3. ELISA

To evaluate the concentrations of human serum amyloid A (SAA1) in plasma samples from AML patients (n = 21) and from healthy donors (n = 15), an enzyme-linked immunosorbent assay (ELISA) was performed according to the corresponding manufacturer’s instructions. SAA1 levels were determined through an ELISA kit (RAB0420, Sigma–Aldrich, St. Louis, MO, USA), with a sensitivity of 500 pg/mL. The values were read at 450 nm in an ELISA reader, and SAA1 concentrations were calculated from specific calibration curves prepared with known standard solutions. Statistical analysis and graphical representations were performed using GraphPad Prism software version 9.0. (GraphPad Software, La Jolla, CA, USA).

### 2.4. Statistical Analysis

All experiments were carried out in triplicate, and the data are expressed as minimum, maximum, median and quartile values. The unpaired Mann–Whitney test was used to compare the results and *p*-value < 0.05 was considered statistically significant (*p* < 0.05). Statistical analyses and graphical representations were performed via GraphPad Prism software version 9.0. (GraphPad Software, La Jolla, CA, USA).

## 3. Results

### 3.1. Bone Marrow Plasma Proteomic Analysis Reveals Differentially Expressed Proteins in AML Patients Compared with Healthy Donors

**Figure 1:**
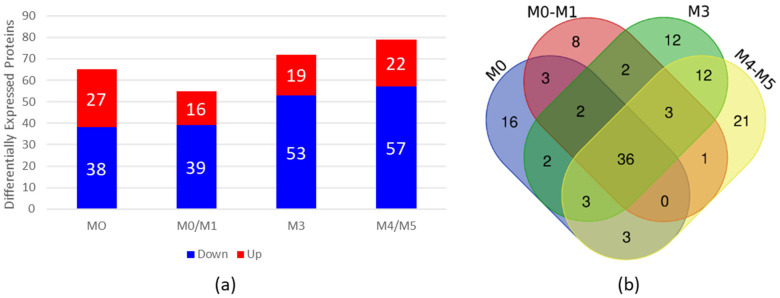
Proteomic analysis of BM plasma from AML patients compared with BM plasma from HDs. (**a**) Differential expression analysis via Expression^E^ showing increased and decreased expression of proteins in BM plasma samples from AML patients compared with BM plasma samples from HDs. The bars in red represent the number of differentially expressed proteins whose expression was increased, and the bars in blue represent the number of differentially expressed proteins whose expression was decreased in the plasma of AML patients. (**b**) Venn diagram of differentially expressed proteins found in each subtype, showing overlapping and unique differentially expressed plasma proteins in each subtype. BM = bone marrow; AML = acute myeloid leukemia; HDs = healthy donors.

To evaluate whether the HN from AML patients is altered, we performed high-throughput proteomic analysis with BM plasma from AML patients and compared it with BM plasma from HDs. For this purpose, we grouped the AML samples into four pools according to the FAB classification (Table 2) and compared them with a pool of HD samples. As a result, we identified differentially expressed (DE) proteins in AML BM plasma samples compared with HD BM plasma samples, of which 65 were DE in the FAB-M0 subtype, 55 in the FAB-M0/M1 subtype, 72 in the FAB-M3 subtype and 79 in the FAB-M4/M5 subtype (Figure 1 and Supplementary Tables S1–S4). Figure 1a shows the number of DE proteins that were overexpressed or downregulated in the four subtypes of AML compared with HDs.

To verify whether the DE proteins found in each subtype had common proteins that could be related to the disease regardless of the subtype, we constructed a Venn diagram http://bioinformatics.psb.ugent.be/webtools/Venn/ (accessed on 6 November 2023) (Figure 1b). Our results revealed 36 common proteins present in all AML BM plasma subtypes in comparison to BM plasma from HDs (Table 3).

### 3.2. Proteins Associated with the Acute Inflammatory Response Are Altered in AML Bone Marrow

To evaluate the signaling pathways related to the 36 common DE proteins identified, we conducted in silico analysis via the KEGG online tool, in which several signaling pathways, including apoptosis, complement and coagulation cascade signaling, metabolic pathways, the MAPK signaling pathway, the PPAR signaling pathway and Rap1, were identified. Many of these pathways have previously been reported to be altered in the context of AML.

We also performed a functional enrichment analysis of the common DE proteins via the online tool WebGestalt, in which several related biological processes were identified; however, only the inflammatory response process was statistically significant, with FDR ≤ 0.05. In this process, the DE proteins C8G, CFB, SAA1, SERPINA3 and SERPINC1 are involved (Table 4).

**Figure 2:**
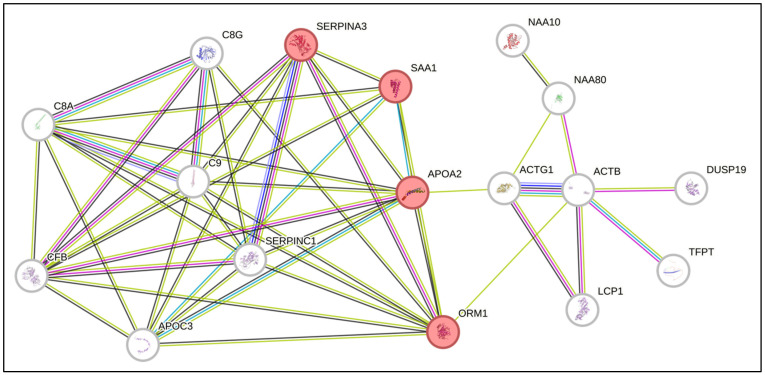
Network of predicted interactions via STRING software (Version 12.0) with the differentially expressed proteins identified in the present study. The proteins highlighted in red are related to the acute inflammatory response.

Furthermore, to verify whether the common proteins interact with each other and with other proteins directly, we performed an analysis via the online tool STRING. Interestingly, we identified interactions among the proteins SAA1, SERPINA3, APOA2 and ORM1, which are involved in the acute inflammatory response process (Figure 2). These proteins have already been described in association with biological processes commonly altered in the context of AML. Interestingly, SAA1 and SERPINA3, which are overexpressed in the proteome of BM marrow from AML patients, are, by nature, secreted proteins and can influence direct signaling to HSCs [22,23].

SERPINA3 is a protein produced by the liver that has proapoptotic activity and is involved in the process of metastatic invasion in melanoma [24]. SAA1 is a secreted protein that is produced in an inflammatory response when tissue damage occurs and is involved in the regulation of the immune response, tissue repair and tumor progression [24]. Studies have shown that SAA1 may play a role in cancer development, tumor evasion, resistance to therapy and progression by promoting metastasis [25,26,27,28]. Thus, considering the role of SAA1 in the inflammatory context, the expression of these proteins as possible serum biomarkers for many tumors [29] and their increased expression in the BM microenvironment of AML patients, we selected SAA1 to confirm its expression via ELISA.

### 3.3. SAA1 Is Altered in AML Bone Marrow Plasma Samples

**Figure 3:**
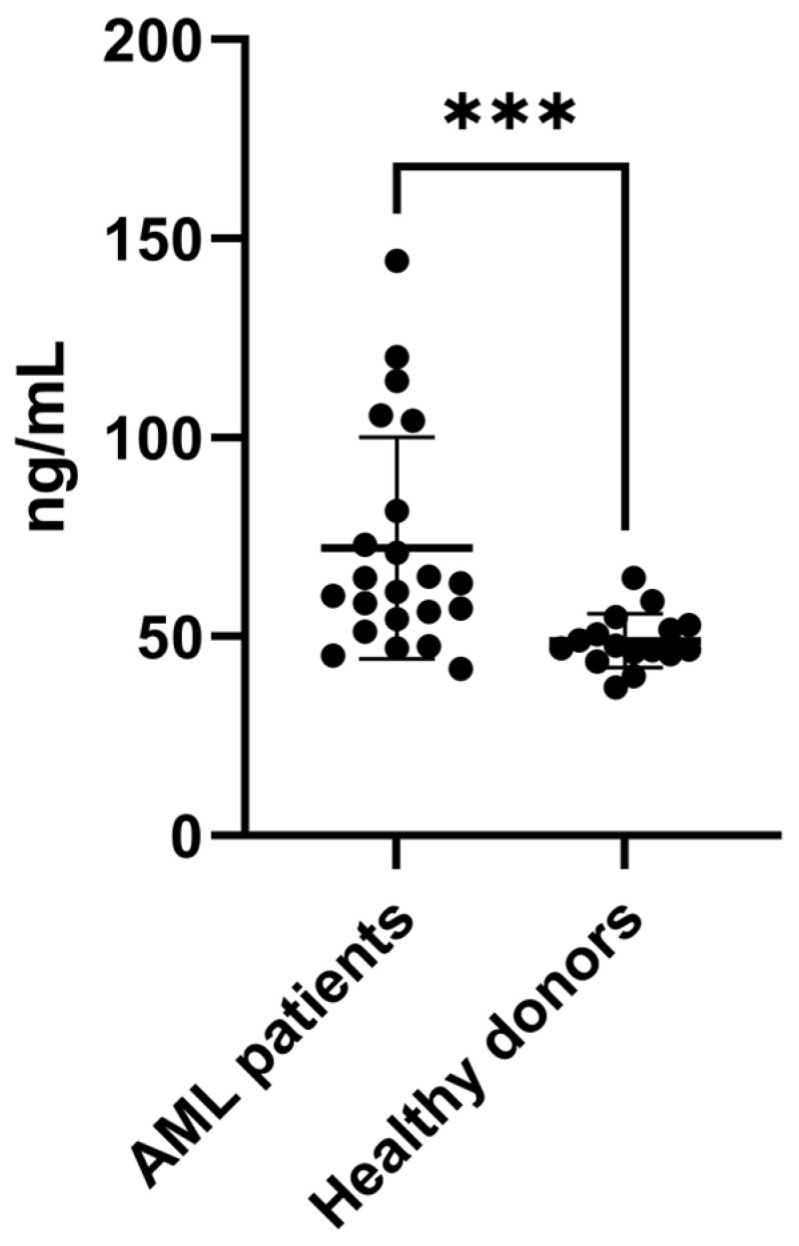
SAA1 is upregulated in plasma samples from AML patients. ELISA was used to evaluate the expression of SAA1 in plasma samples from AML patients (n = 22) and healthy donors (n = 16). The results revealed that SAA1 protein expression is increased in BM plasma from AML patients. Boxplot of individual values representing the minimum, maximum, median and quartile values of SAA1 plasma concentration. *** *p* < 0.001. AML = acute myeloid leukemia.

To confirm the increased expression of SAA1 proteins in BM plasma from AML patients, we performed an ELISA with a larger number of samples, including samples from patients with different AML subtypes (n = 22) and healthy donors (n = 16) (Table 1 and Table 2—ELISA cohort). As shown in Figure 3, the results confirmed the increase in SAA1 expression in AML plasma samples, corroborating the proteomic approach. Taken together, these results showed that SAA1 is overexpressed in BM plasma samples from AML patients regardless of the FAB subtype or risk classification, indicating that this protein could be a good candidate for an AML biomarker.

### 3.4. SAA1 and Immune Signaling Pathways

To identify the role of SAA1 in signaling pathways, we performed an in silico analysis via the PathwayBrowser tool in the Reactome Pathway Database. Interestingly, our results revealed that SAA1 is capable of stimulating immune signaling through the activation of NF-kappa-B (NF-κB). This association has been previously confirmed in other contexts, such as in human and murine intestinal epithelial cells, indicating that SAA1 potentially participates in the inflammatory process [30,31].

## 4. Discussion

Acute myeloid leukemia (AML) presents a significant clinical challenge because of its heterogeneous nature and complex genetic landscape. Despite advancements in understanding its pathogenesis and treatment, accurately diagnosing AML remains difficult, necessitating the identification of reliable biomarkers. In this sense, proteomics can help to identify diagnostic biomarkers of AML. Recently, many potential protein biomarkers have been reported in the literature for the diagnosis of AML, e.g., complement factor C7, complement factor H, HPT (Haptoglobin), ApoE (Apolipoprotein E), plasminogen and ApoA1 (Apolipoprotein A1), via proteomics techniques [13,32,33,34]. Therefore, in the present study, we used high-throughput proteomics to identify common biomarkers in the HN of AML patients, regardless of risk classification and disease subtype. The technique used is capable of screening and identifying the protein profile of biological fluids, including BM plasma, with the aim of discovering biomarkers to diagnose early AML, predict prognosis, determine therapeutic efficacy, identify novel drug targets, and ultimately develop personalized medicine [35].

Proteomic analysis revealed alterations in the plasma protein profile from the BM of AML patients compared with that of healthy donors, and in this analysis, we identified 36 proteins that were differentially expressed across all AML FAB subtypes. The characterization of these common differentially expressed proteins is important, as it contributes to the understanding of AML biology and altered signaling in the bone marrow microenvironment, as well as the identification of markers that can be found in all patients with this disease.

Among the 36 common proteins identified, although only 8.3% (3/36) of the proteins presented increased expression, they are related to biological processes frequently altered in AML, including acute inflammation, which was recently described to be altered in the plasma of AML patients. In the context of AML, inflammation has been linked to very early stages of the disease, starting with a premalignant condition known as clonal hematopoiesis, where blood cells carrying specific AML-related mutations accumulate in the blood. However, the role of inflammation in altering the immune microenvironment of AML remains unclear [36]. Interaction analysis revealed that SAA1 and SERPINA3 are involved in the acute inflammatory response, suggesting a coordinated role in AML pathogenesis.

The highly expressed SERPINA3 (Serpin Family A Member 3) protein is produced by the liver, has proapoptotic activity and is involved in the process of metastatic invasion in melanoma [23]. SERPINA3 may be regulated through the JAK-STAT signaling pathway, which is commonly altered in AML [37].

SAA1, a member of the apolipoprotein family, is a secreted proinflammatory protein that plays critical roles in immune regulation and tissue repair [38]. SAA promotes myeloid cell survival during infection and inflammation [39] and is involved in the regulation of tumor progression [26]. Studies have shown that SAA may play a role in cancer development and progression by promoting metastasis. SAA1 can also modulate the immune response, potentially leading to tumor evasion and therapy resistance [25,26,27,28]. High levels of SAA1 in the blood or tumor tissue have been associated with poor prognosis in patients with lung cancer, gastric cancer, endometrial cancer, prostate cancer, melanoma and esophageal cancer and have been suggested as potential biomarkers for several types of cancer [40]. Takehara et al. demonstrated that SAA1 promotes cancer progression in a pancreatic cancer cell line and that silencing SAA1 could decrease their migration and chemoresistance [41].

In the context of hematopoiesis, Stavrou et al. reported that SAA1 promotes AML blast viability and that elevated SAA1 levels are associated with a negative impact on HSCs from AML patients [42,43]. SAA1 also selectively promotes the growth of patient-derived AML cells independent of the cytogenetic or mutational profile. In AML, the SAA1 protein is secreted by osteoblasts and is capable of inducing a pro-proliferative effect on AML blasts and leads to the self-reinforced progression of leukemia [43,44]. The overexpression of SLPI by osteoblasts also indicates the role of SAA1 signaling in HSCs, since osteoblasts are recognized as necessary for the maintenance and control of the number and function of HSCs in the hematopoietic niche.

Considering the importance of SAA1 in HN and AML, with the aim of its use as a potential diagnostic biomarker for AML, we confirmed the increased expression of SAA1 through an ELISA with a large cohort of plasma samples from AML patients with different subtypes compared with a group of plasma samples from healthy donors. Raza and colleagues, using a multidimensional fractionation strategy of pooled plasma samples from AML patients in comparison with those from healthy controls, reported an increase in SAA1 expression, and this protein could be used as a potential biomarker for AML [45]. This consistency between different analytical methods confirms the elevated expression of SAA1 and reinforces the possible use of SAA1 as a diagnostic biomarker for AML, independent of subtype classification.

To understand the impact of increased SAA1 expression in the BM microenvironment of patients with AML, our in silico results revealed that SAA1 could activate inflammatory signaling pathways in other tumors and that these pathways are associated with cell invasion, metastasis, migration and drug resistance by activating the transcription factor nuclear factor kappa B (NF-κB) [46]. NF-κB is a regulator of many biological functions, such as cell survival, apoptosis, invasion and hematopoiesis, where it regulates HSC self-renewal and differentiation in myeloid and lymphoid lineages. It is known that NF-κB is constitutively activated in the majority of AML patients, which promotes cell survival and resistance to therapy by activating antiapoptotic genes and promoting inflammation [47]. Baumgartner and colleagues reported that NF-κB signaling is constitutively active in CD34+ stem cells from M3, M4 and M5 AML patients [48]. In addition, researchers have developed blocking monoclonal antibodies against SAA1 as promising novel targets for the treatment of hematological malignancies to inhibit SAA1’s potent oncogenic effects on malignant HSCs and to examine its potential therapeutic activity. In their findings, an antibody was able to inhibit 85% of SAA1-mediated NFκB signaling [49].

In human amnion fibroblasts, it was described that by binding to receptor Toll-like 4 (TLR4), secreted SAA1 activates ERK1/2 (Extracellular Signal-Regulated Kinases 1/2) and p38, which increase not only the phosphorylation of p65 but also the degradation of IκB (Inhibitor of Kappa B), resulting in the activation of NFκB. Activated NFκB induces the expression of proinflammatory genes including *IL-1β (Interleukin-1 Beta)*, *IL-6 (Interleukin-6)* and *COX-2 (Cyclooxygenase-2)*, the rate-limiting enzyme in *PGE2 (Prostaglandin E2)* synthesis. Further studies should be conducted to evaluate the impact of increased SAA1 on the NF-κB pathway in the bone marrow microenvironment of AML patients, particularly in hematopoietic stem cells, and its relationship with the leukemic transformation process [29].

The accumulated knowledge on AML over the decades shows that numerous genetic alterations have been described and are already tested at diagnosis. Even so, based on classification systems, flow cytometry remains widely implemented in diagnostics, essential for identifying and enumerating leukemic blasts, assigning lineage and detecting aberrant immunophenotypic features. In association, cytogenetic analyses are performed to detect chromosomal alterations, some of which have diagnostic value, such as t(8;21) and t(15;17), and molecular biology analyses can identify mutations in *FLT3 (FMS-Like Tyrosine Kinase 3)*, *NPM1 (Nucleophosmin 1)* and *CEBPA (CCAAT Enhancer-Binding Protein Alpha)*, with prognostic significance [6].

However, the current gold standard for AML diagnosis includes NGS (Next-Generation Sequencing) analysis of approximately 40 different mutations, including germline mutations and various other translocations. Still, many of these alterations do not yet have a well-established diagnostic or prognostic role. Nonetheless, the results obtained by all these analyses contribute to expanding knowledge and improving patient stratification to enable more individualized treatments. In this context, SAA1 could serve as an additional diagnostic biomarker, as we observed increased expression in the analyzed patients. However, to evaluate and confirm the “power” and potential use of SAA1 as a diagnostic biomarker in clinical practice, further studies with a larger and more diverse sample size are essential [6].

These findings position SAA1 as a promising biomarker for AML diagnosis, offering a potential tool for more accurate identification of the disease. Nevertheless, further research should explore the mechanisms by which SAA1 influences the process of leukemic transformation in AML and its potential clinical use to improve outcomes for patients with this disease.

## Figures and Tables

**Table 1:** List of healthy donors who participated in this study.

Code Lab	Sex	Age (Years)	Proteomic Cohort	ELISA * Cohort
001/16	Female	25	X	
002/16	Female	32	X	
003/16	Male	23	X	
004/16	Female	30	X	
005/16	Male	28	X	
014/17	Female	26		X
015/17	Female	58		X
016/17	Female	60		X
078/19	Male	44		X
093/19	Female	44		X
097/19	Female	46		X
098/19	Male	22		X
100/19	Male	34		X
104/19	Male	29		X
113/20	Female	43		X
002/21	Female	52		X
005/21	Male	46		X
003/22	Male	60		X
001/23	Female	37		X
005/23	Male	41		X
001/24	Male	34		X

* ELISA = enzyme-linked immunosorbent assay.

**Table 2:** List of AML patients who participated in this study.

Code Lab	Sex	Age (Years)	FAB Classification	Genetic Abnormality	Risk Category *	Proteomic Cohort	ELISA Cohort
002/12	Male	68	M0/M1	Normal karyotype	Favorable	X	
003/12	Female	82	M1/M2	47Xdel(X)(q23)+i(11)(q10)[13]/46,XX[15]	Intermediate		X
015/12	Female	44	M2/M3	Unknown	Unknown		X
017/12	Female	66	M0	Unknown	Unknown	X	X
011/13	Male	35	M4/M5	Normal karyotype, Mutated FLT3	Favorable	X	
017/13	Male	25	M3	Unknown	Unknown		X
025/13	Male	38	M3	Normal karyotype	Favorable	X	
033/13	Female	51	M4/M5	Normal karyotype	Favorable	X	
053/14	Male	35	M1	Unknown	Unknown		X
001/16	Female	43	M4/M5	46XX, +(3;19)(p22;p13);(t9:22)(q34 q11);del (13)(q11: q20)[20]	Adverse	X	
007/16	Male	22	M3	PML::RARA fusion	Intermediate	X	
008/16	Female	68	M3	PML::RARA fusion	Intermediate	X	
013/16	Female	80	M0/M1	47,XX,+[8]/47,XX,del(X)(q22),+8[13]/46,XX[1]	Adverse	X	X
018/16	Male	37	M3	PML::RARA fusion	Intermediate	X	
020/16	Male	64	M2	Unknown	Unknown		X
028/16	Female	40	M0	Normal karyotype	Favorable		X
029/16	Male	23	M2	t(9;11)(p21.3;q23.3)/MLLT3::KMT2A	Intermediate		X
031/16	Male	53	M0/M1	Normal karyotype	Favorable	X	X
045/17	Male	51	M0/M1	Unknown	Unknown		X
049/17	Female	60	M3	t(15;17)(q24.1;q21.2)/PML::RARE	Intermediate		X
056/17	Female	65	M2	Unknown	Unknown		X
058/17	Female	53	M2	t(15;17)(q24.1;q21.2)/PML::RARE	Intermediate		X
001/18	Male	35	M4/M5	Normal karyotype	Favorable		X
004/18	Female	39	M3	PML::RARA fusion	Intermediate		X
001/19	Male	20	M2	t(2:14)(q22:q31)	Intermediate		X
006/20	Male	73	M4	Normal karyotype	Favorable		X
003/21	Female	21	M4	inv(16)(p13.1q22) or t(16;16)(p13.1;q22)/CBFB::MYH11	Intermediate		X
008/22	Female	31	M3	Unknown	Unknown		X
001/24	Female	69	M2	Unknown	Unknown		X
003/24	Female	58	M4	Unknown	Unknown		X

* 2022 ELN risk classification by genetic at initial diagnosis. ELISA = enzyme-linked immunosorbent assay.

**Table 3:** The 36 common differentially expressed proteins present in all AML BM plasma samples.

Accession Code	Symbol	Description	Expression
P02656	APOC3	Apolipoprotein C-III	Increased
P0DJI8	SAA1	Serum amyloid A-1 protein	Increased
P01011	SERPINA3	Alpha-1-antichymotrypsin	Increased
P0DP03	IGHV3-30-5	Immunoglobulin heavy variable 3-30-5	Decreased
P07360	C8G	Complement component C8 gamma chain	Decreased
Q9GZL8	BPESC1	Putative BPES syndrome breakpoint region protein	Decreased
Q2T9K0	TMEM44	Transmembrane protein 44 OS=Homo sapiens	Decreased
P0DP02	IGHV3-30-3	Immunoglobulin heavy variable 3-30-3	Decreased
Q9BV90	SNRNP25	U11/U12 small nuclear ribonucleoprotein 25 kDa protein	Decreased
P00751	CFB	Complement factor B OS=Homo sapiens	Decreased
Q4LEZ3	AARD	Alanine and arginine-rich domain-containing protein	Decreased
P54284	CACNB3	Voltage-dependent L-type calcium channel subunit beta-3	Decreased
P21453	S1PR1	Sphingosine 1-phosphate receptor 1	Decreased
P13796	LCP1	Plastin-2 OS=Homo sapiens	Decreased
P0C1Z6	TFPT	TCF3 fusion partner	Decreased
P46926	GNPDA1	Glucosamine-6-phosphate isomerase 1	Decreased
A0A075B6N3	TRBV24-1	T cell receptor beta variable 24-1	Decreased
P01772	IGHV3-33	Immunoglobulin heavy variable 3-33	Decreased
P60709	ACTB	Actin_ cytoplasmic 1	Decreased
P01008	SERPINC1	Antithrombin-III	Decreased
P01768	IGHV3-30	Immunoglobulin heavy variable 3-30	Decreased
P41227	NAA10	N-alpha-acetyltransferase 10	Decreased
P01767	IGHV3-53	Immunoglobulin heavy variable 3-53	Decreased
A0A0C4DH42	IGHV3-66	Immunoglobulin heavy variable 3-66	Decreased
Q9Y3Q3	TMED3	Voltage-dependent L-type calcium channel subunit beta-3	Decreased
Q8WTR2	DUSP19	Dual specificity protein phosphatase 19	Decreased
P62324	BTG1	Protein BTG1 OS=Homo sapiens	Decreased
Q03403	TFF2	Trefoil factor 2 OS=Homo sapiens	Decreased
P01764	IGHV3-23	Immunoglobulin heavy variable 3-23	Decreased
Q9Y600	CSAD	Cysteine sulfinic acid decarboxylase	Decreased
Q86WV1	SKAP1	Src kinase-associated phosphoprotein 1	Decreased
Q6UXB4	CLEC4G	C-type lectin domain family 4 member G	Decreased
P63261	ACTG1	Actin_cytoplasmic 2 OS=Homo sapiens	Decreased
Q6ZTI0	DKFZ	Putative uncharacterized protein FLJ44636	Decreased
Q86VE3	SATL1	Spermidine/spermine N(1)-acetyltransferase-like protein 1	Decreased
Q8N6L0	CCDC155	Protein KASH5 OS=Homo sapiens	Decreased

**Table 4:** Gene Ontology terms associated with the 36 common differentially expressed proteins in HNs from AML patients compared with BM from HDs.

Overrepresentation Analysis	Common Differentially Expressed Proteins			
	Up *	Down *	FDR	Database
Acute inflammatory response	SAA1, SERPINA3	C8G, CFB, SERPINC1	0.004	GOBP (GO:0002526)
Protein activation cascade		C8G, CFB, SERPINC1	0.148	GOBP (GO:0072376)
Anatomical structure homeostasis	SERPINA3	S1PR1, ACTB, TFF2, ACTG1	0.148	GOBP (GO:0060249)
Multicellular organismal homeostasis	SERPINA3	S1PR1, ACTB, TFF2, ACTG1	0.210	GOBP (GO:0048871)
Coagulation	SAA1	ACTB, SERPINC1, ACTG1	0.344	GOBP (GO:0050817)
Positive chemotaxis	SAA1	S1PR1	0.708	GOBP (GO:0050918)
Homotypic cell–cell adhesion		ACTB, ACTG1	0.708	GOBP (GO:0034109)
Ephrin receptor signaling pathway		ACTB, ACTG1	0.708	GOBP (GO:0048013)
Regulation of body fluid levels	SAA1	ACTB, SERPINC1, ACTG1	0.708	GOBP (GO:0050878)
Immune response-regulating signaling pathway		CACNB3, ACTB, SKAP1, ACTG1	0.708	GOBP (GO:0002764)

* The up and down proteins refer to the expression of these proteins in BM from AML patients in comparison with the expression of BM from healthy donors.

## Data Availability

The original contributions presented in this study are included in the article and Supplementary Materials. Further inquiries can be directed to the corresponding author.

## Supplementary Materials

The following supporting information can be downloaded at https://www.mdpi.com/article/10.3390/biomedicines13040880/s1, Table S1: List of the 65 differentially expressed proteins identified in AML BM plasma from FAB-M0 subtype patients compared with HD BM plasma samples; Table S2: List of the 55 differentially expressed proteins identified in AML BM plasma from FAB-M0-M1 subtype patients compared with HD BM plasma samples; Table S3: List of the 72 differentially expressed proteins identified in AML BM plasma from FAB-M3 subtype patients compared with HD BM plasma samples; Table S4: List of the 79 differentially expressed proteins identified in AML BM plasma from FAB-M4-M5 subtype patients compared with HD BM plasma samples.

## Abbreviations

The following abbreviations are used in this manuscript:

AML	Acute myeloid leukemia
BM	Bone marrow
HN	Hematopoietic niche
HD	Healthy donors
DE	Differentially expressed
HSC	Hematopoietic stem cell

## References

[1] Döhner H., Weisdorf D.J., Bloomfield C.D., Leukemia A.M. (2015). Acute Myeloid Leukemia. N. Engl. J. Med..

[2] Zhou Y., Huang G., Cai X., Liu Y., Qian B., Li D. (2024). Global, regional, and national burden of acute myeloid leukemia, 1990–2021: A systematic analysis for the global burden of disease study 2021. Biomark. Res..

[3] Becker M., Farina K.A., Mascarenhas J. (2024). Acute myeloid leukemia. J. Am. Acad. Physician Assist..

[4] Bennett J.M., Catovsky D., Daniel M.T., Flandrin G., Galton D.A., Gralnick H.R., Sultan C. (1985). Proposed revised criteria for the classification of acute myeloid leukemia. A report of the French-American-British Cooperative Group. Ann. Intern. Med..

[5] Bennett J.M., Catovsky D., Daniel M.-T., Flandrin G., Galton D.A.G., Gralnick H.R., Sultan C. (1976). Proposals for the Classification of the Acute Leukaemias French-American-British (FAB) Co-operative Group. Br. J. Haematol..

[6] Döhner H., Wei A.H., Appelbaum F.R., Craddock C., DiNardo C.D., Dombret H., Ebert B.L., Fenaux P., Godley L.A., Hasserjian R.P. (2022). Diagnosis and Management of AML in Adults: 2022 ELN Recommendations from an International Expert Panel. Blood.

[7] Kim N., Hahn S., Choi Y.J., Cho H., Chung H., Jang J.E., Lyu C.J., Lee S.-T., Choi J.R., Cheong J.-W. (2024). Comprehensive insights into AML relapse: Genetic mutations, clonal evolution, and clinical outcomes. Cancer Cell Int..

[8] Lapidot T., Sirard C., Vormoor J., Murdoch B., Hoang T., Caceres-Cortes J., Minden M., Paterson B., Caligiuri M.A., Dick J.E. (1994). A cell initiating human acute myeloid leukaemia after transplantation into SCID mice. Nature.

[9] Dick J.E. (2005). Acute myeloid leukemia stem cells. Ann. N. Y. Acad. Sci..

[10] Zhang P., Zhang C., Li J., Han J., Liu X., Yang H. (2019). The physical microenvironment of hematopoietic stem cells and its emerging roles in engineering applications. Stem Cell Res. Ther..

[11] Pimenta D.B., Varela V.A., Datoguia T.S., Caraciolo V.B., Lopes G.H., Pereira W.O. (2021). The Bone Marrow Microenvironment Mechanisms in Acute Myeloid Leukemia. Front. Cell Dev. Biol..

[12] Ota J., Yamashita Y., Okawa K., Kisanuki H., Fujiwara S., Ishikawa M., Choi Y.L., Ueno S., Ohki R., Koinuma K. (2003). Proteomic analysis of hematopoietic stem cell-like fractions in leukemic disorders. Oncogene.

[13] Kwak J.-Y., Ma T.-Z., Yoo M.-J., Choi B.H., Kim H.-G., Kim S.-R., Yim C.-Y., Kwak Y.-G. (2004). The comparative analysis of serum proteomes for the discovery of biomarkers for acute myeloid leukemia. Exp. Hematol..

[14] Balkhi M.Y., Trivedi A.K., Geletu M., Christopeit M., Bohlander S.K., Behre H.M., Behre G. (2006). Proteomics of acute myeloid leukaemia: Cytogenetic risk groups differ specifically in their proteome, interactome and post-translational protein modifications. Oncogene.

[15] Strassberger V., Gutbrodt K.L., Krall N., Roesli C., Takizawa H., Manz M.G., Fugmann T., Neri D. (2014). A comprehensive surface proteome analysis of myeloid leukemia cell lines for therapeutic antibody development. J. Proteom..

[16] Chernak B., Galan-Diez M., Ali A.M., Cuesta-Dominguez A., Chen Z., Koehnke T., Majeti R., Carroll M., Raza A., Kousteni S. (2023). Serum Amyloid A1 (SAA1) Secreted By the Stromal Microenvironment Drives Malignant Clonal Proliferation in Myelodysplastic Syndromes (MDS) and Acute Myeloid Leukemia (AML). Blood.

[17] Khoury J.D., Solary E., Abla O., Akkari Y., Alaggio R., Apperley J.F., Bejar R., Berti E., Busque L., Chan J.K.C. (2022). The 5th edition of the World Health Organization Classification of Haematolymphoid Tumours: Myeloid and Histiocytic/Dendritic Neoplasms. Leukemia.

[18] Bradford M.M. (1976). Determinacion de Proteinas: Metodo de Bradford. Anal. Biochem..

[19] Pizzatti L., Panis C., Lemos G., Rocha M., Cecchini R., Souza G.H.M.F., Abdelhay E. (2012). Label-free MS E proteomic analysis of chronic myeloid leukemia bone marrow plasma: Disclosing new insights from therapy resistance. Proteomics.

[20] Panis C., Pizzatti L., Herrera A.C., Corrêa S., Binato R., Abdelhay E. (2014). Label-free proteomic analysis of breast cancer molecular subtypes. J. Proteome Res..

[21] Corrêa S., Panis C., Binato R., Herrera A.C., Pizzatti L., Abdelhay E. (2017). Identifying potential markers in Breast Cancer subtypes using plasma label-free proteomics. J. Proteom..

[22] Wei Y., Bueso-Ramos C.E., Yang H., Jia Y., Zheng H., Colla S., Nguyen M., Fernandez M., Kantarjian H.M., Garcia-Manero G. (2012). Serum Amyloid Protein A 1 (hSAA1) Is Overexpressed in Myelodysplastic Syndromes and Potentially Mediates Toll-Like Receptor 2 Innate Immunity Signaling in CD34+ Hematopoietic Stem Cells. Blood.

[23] de Mezer M., Rogaliński J., Przewoźny S., Chojnicki M., Niepolski L., Sobieska M., Przystańska A. (2023). SERPINA3: Stimulator or Inhibitor of Pathological Changes. Biomedicines.

[24] Zhou J., Cheng Y., Tang L., Martinka M., Kalia S. (2017). Up-regulation of SERPINA3 correlates with high mortality of melanoma patients and increased migration and invasion of cancer cells. Oncotarget.

[25] Wang X., Wen S., Du X., Zhang Y., Yang X., Zou R., Feng B., Fu X., Jiang F., Zhou G. (2023). SAA suppresses α-PD-1 induced anti-tumor immunity by driving TH2 polarization in lung adenocarcinoma. Cell Death Dis..

[26] Niu X., Yin L., Yang X., Yang Y., Gu Y., Sun Y., Yang M., Wang Y., Zhang Q., Ji H. (2022). Serum amyloid A 1 induces suppressive neutrophils through the Toll-like receptor 2–mediated signaling pathway to promote progression of breast cancer. Cancer Sci..

[27] Zhao Y., Chen Y., Wan Q., Xiao C., Guo Z., Du X., Hu Y., Zheng A., Cao Z. (2023). Identification of SAA1 as a novel metastasis marker in ovarian cancer and development of a graphene-based detection platform for early assessment. J. Cancer Res. Clin. Oncol..

[28] Li Z., Hou Y., Zhao M., Li T., Liu Y., Chang J., Ren L. (2020). Serum amyloid a, a potential biomarker both in serum and tissue, correlates with ovarian cancer progression. J. Ovarian Res..

[29] Sack G.H. (2018). Serum amyloid A—A review. Mol. Med..

[30] Li W., Wang W., Zuo R., Liu C., Shu Q., Ying H., Sun K. (2017). Induction of pro-inflammatory genes by serum amyloid A1 in human amnion fibroblasts. Sci. Rep..

[31] Chen D.-W., Fan J.-M., Schrey J.M., Mitchell D.V., Jung S.K., Hurwitz S.N., Perez E.B., Muraro M.J., Carroll M., Taylor D.M. (2024). Inflammatory recruitment of healthy hematopoietic stem and progenitor cells in the acute myeloid leukemia niche. Leukemia.

[32] Lee S., Kim I.J., Jeong B.Y., Choi M., Kim J.Y., Kwon K., Lee J.W., Yu A., Shin M. (2012). Use of MDLC-DIGE and LC-MS/MS to identify serum biomarkers for complete remission in patients with acute myeloid leukemia. Electrophoresis.

[33] Braoudaki M., Tzortzatou-Stathopoulou F., Anagnostopoulos A.K., Papathanassiou C., Vougas K., Karamolegou K., Tsangaris G.T. (2011). Proteomic analysis of childhood de novo acute myeloid leukemia and myelodysplastic syndrome/AML: Correlation to molecular and cytogenetic analyses. Amino Acids.

[34] Zheng R., Ma X. (2013). Study on serum protein mass spectrometric characteristics of acute leukemia. Zhonghua Xue Ye Xue Za Zhi.

[35] Xiang W., Lam Y.H., Periyasamy G., Chuah C. (2022). Application of High Throughput Technologies in the Development of Acute Myeloid Leukemia Therapy: Challenges and Progress. Int. J. Mol. Sci..

[36] Lasry A., Nadorp B., Fornerod M., Nicolet D., Wu H., Walker C.J., Sun Z., Witkowski M.T., Tikhonova A.N., Guillamot-Ruano M. (2023). An inflammatory state remodels the immune microenvironment and improves risk stratification in acute myeloid leukemia. Nat. Cancer.

[37] Moser B., Edtmayer S., Witalisz-Siepracka A., Stoiber D. (2021). The Ups and Downs of STAT Inhibition in Acute Myeloid Leukemia. Biomedicines.

[38] Lowell C.A., Stearman R.S., Morrow J.F. (1986). Transcriptional regulation of serum amyloid A gene expression. J. Biol. Chem..

[39] Sander L.E., Sackett S.D., Dierssen U., Beraza N., Linke R.P., Müller M., Blander J.M., Tacke F., Trautwein C. (2010). Hepatic acute-phase proteins control innate immune responses during infection by promoting myeloid-derived suppressor cell function. J. Exp. Med..

[40] Lin H., Tan G., Liu Y., Lin S. (2019). The prognostic value of serum amyloid A in solid tumors: A meta-analysis. Cancer Cell Int..

[41] Jajula S., Naik V., Kalita B., Yanamandra U., Sharma S., Chatterjee T., Bhanuse S., Bhavsar P.P., Taunk K., Rapole S. (2024). Integrative proteome analysis of bone marrow interstitial fluid and serum reveals candidate signature for acute myeloid leukemia. J. Proteom..

[42] Stavrou V., Fultang L., Booth S., De Simone D., Bartnik A., Scarpa U., Gneo L., Panetti S., Potluri S., Almowaled M. (2023). Invariant NKT cells metabolically adapt to the acute myeloid leukaemia environment, Cancer Immunology. Immunotherapy.

[43] Galán-Díez M., Borot F., Ali A.M., Zhao J., Gil-Iturbe E., Shan X., Luo N., Liu Y., Huang X.-P., Bisikirska B. (2022). Subversion of Serotonin Receptor Signaling in Osteoblasts by Kynurenine Drives Acute Myeloid Leukemia. Cancer Discov..

[44] Allert C., Müller-Tidow C., Blank M.F. (2024). The relevance of the hematopoietic niche for therapy resistance in acute myeloid leukemia. Int. J. Cancer.

[45] Raza S.K., Saleem M., Shamsi T., Choudhary M.I., Rahman A.U., Musharraf S.G. (2017). 5D proteomic approach for the biomarker search in plasma: Acute myeloid leukaemia as a case study. Sci. Rep..

[46] Lin Y., Bai L., Chen W., Xu S. (2010). The NF-κB activation pathways, emerging molecular targets for cancer prevention and therapy. Expert. Opin. Ther. Targets.

[47] Di Francesco B., Verzella D., Capece D., Vecchiotti D., Di Vito Nolfi M., Flati I., Cornice J., Di Padova M., Angelucci A., Alesse E. (2022). NF-κB: A Druggable Target in Acute Myeloid Leukemia. Cancers.

[48] Baumgartner B., Weber M., Quirling M., Fischer C., Page S., Adam M., von Schilling C., Waterhouse C., Schmid C., Neumeier D. (2002). Increased IκB kinase activity is associated with activated NF-κB in acute myeloid blasts. Leukemia.

[49] Abbas H.A., Hao D., Tomczak K., Barrodia P., Im J.S., Reville P.K., Alaniz Z., Wang W., Wang R., Wang F. (2020). Single-Cell Characterization of Acute Myeloid Leukemia (AML) and Its Microenvironment Identifies Signatures of Resistance to PD-1 Blockade Based Therapy. Blood.

