# Supplementary material for: SAA1 Protein: A Potential Biomarker for Acute Myeloid Leukemia
Source: Biomedicines. 2025 Apr 5;13(4):880. doi: 10.3390/biomedicines13040880 (PMC12024993; doi:10.3390/biomedicines13040880)
Supplement: Supplementary file 1 [file biomedicines-13-00880-s001.zip › biomedicines-3502395-supplementary.pdf]

biomedicines

MDPI

**Table S1.** List of the 65 differentially expressed proteins identified in AML BM plasma from FAB-M0 subtype patients compared with HD BM plasma samples.

| Accession Code | Symbol | Description | Expression |
|---|---|---|---|
| **P02042** | HBD | Hemoglobin subunit delta | Decreased |
| **P68871** | HBB | Hemoglobin subunit beta | Decreased |
| **P00738** | HP | Haptoglobin | Decreased |
| **P00739** | HPR | Haptoglobin-related protein | Decreased |
| **P02749** | APOH | Beta-2-glycoprotein 1 | Decreased |
| **A0M8Q6** | IGLC7 | Immunoglobulin lambda constant 7 | Decreased |
| **P0DOX7** | IGK_HUMAN | Immunoglobulin kappa light chain | Decreased |
| **P01834** | IGKC | Immunoglobulin kappa constant | Decreased |
| **P02679** | FGG | Fibrinogen gamma chain | Decreased |
| **P02675** | FGB | Fibrinogen beta chain | Decreased |
| **P02671** | FGA | Fibrinogen alpha chain | Decreased |
| **P60709** | ACTB | Actin_ cytoplasmic 1 | Decreased |
| **P63261** | ACTG1 | Actin_ cytoplasmic 2 | Decreased |
| **Q4LEZ3** | AARD | Alanine and arginine-rich domain-containing protein | Decreased |
| **P01008** | SERPINC1 | Antithrombin-III | Decreased |
| **P07360** | C8G | Complement component C8 gamma chain | Decreased |
| **P00751** | CFB | Complement factor B OS=Homo sapiens | Decreased |
| **Q6UXB4** | CLEC4G | C-type lectin domain family 4 member G | Decreased |
| **Q9Y600** | CSAD | Cysteine sulfinic acid decarboxylase | Decreased |
| **Q8WTR2** | DUSP19 | Dual specificity protein phosphatase 19 | Decreased |
| **P46926** | GNPDA1 | Glucosamine-6-phosphate isomerase 1 | Decreased |
| **P01764** | IGHV3-23 | Immunoglobulin heavy variable 3-23 | Decreased |
| **P01768** | IGHV3-30 | Immunoglobulin heavy variable 3-30 | Decreased |
| **P0DP02** | IGHV3-30-3 | Immunoglobulin heavy variable 3-30-3 | Decreased |
| **P0DP03** | IGHV3-30-5 | Immunoglobulin heavy variable 3-30-5 | Decreased |
| **P01772** | IGHV3-33 | Immunoglobulin heavy variable 3-33 | Decreased |
| **P01767** | IGHV3-53 | Immunoglobulin heavy variable 3-53 | Decreased |
| **A0A0C4DH42** | IGHV3-66 | Immunoglobulin heavy variable 3-66 | Decreased |
| **P01624** | IGKV3-15 | Immunoglobulin kappa variable 3-15 | Decreased |
| **A0A087WSY6** | IGKV3D-15 | Immunoglobulin kappa variable 3D-15 | Decreased |
| **P41227** | NAA10 | N-alpha-acetyltransferase 10 | Decreased |
| **P13796** | LCP1 | Plastin-2 | Decreased |
| **P62324** | BTG1 | Protein BTG1 | Decreased |
| **Q8N6L0** | CCDC155 | Protein KASH5 | Decreased |
| **Q9GZL8** | BPESC1 | Putative BPES syndrome breakpoint region protein | Decreased |
| **Q8N8H1** | ZNF321P | Putative protein ZNF321 | Decreased |
| **Q6ZTI0** | YK032 | Putative uncharacterized protein | Decreased |
| **Q86VE3** | SATL1 | Spermidine/spermine N(1)-acetyltransferase-like protein 1 | Decreased |
| **P21453** | S1PR1 | Sphingosine 1-phosphate receptor 1 | Decreased |
| **Q86WV1** | SKAP1 | Src kinase-associated phosphoprotein 1 | Decreased |
| **A0A075B6N3** | TRBV24-1 | T cell receptor beta variable 24-1 | Decreased |
| **P0C1Z6** | TFPT | TCF3 fusion partner | Decreased |
| **Q9Y3Q3** | TMED3 | Transmembrane emp24 domain-containing protein 3 | Decreased |
| **Q2T9K0** | TMEM44 | Transmembrane protein 44 | Decreased |
| **Q03403** | TFF2 | Trefoil factor 2 OS=Homo sapiens | Decreased |
| **Q9BV90** | SNRNP25 | U11/U12 small nuclear ribonucleoprotein 25 kDa protein | Decreased |

 https://doi.org/10.3390/biomedicines13040880

| | | | |
|---|---|---|---|
| **P54284** | CACNB3 | Voltage-dependent L-type calcium channel subunit beta-3 | Decreased |
| **P14550** | AKR1A1 | Aldo-keto reductase family 1 member A1 | Increased |
| **P01011** | SERPINA3 | Alpha-1-antichymotrypsin | Increased |
| **P08697** | SERPINF2 | Alpha-2-antiplasmin | Increased |
| **Q8WVL7** | ANKRD49 | Ankyrin repeat domain-containing protein 49 | Increased |
| **P02656** | APOC3 | Apolipoprotein C-III | Increased |
| **P01031** | C5 | Complement C5 | Increased |
| **P02751** | FN1 | Fibronectin | Increased |
| **Q03014** | HHEX | Hematopoietically-expressed homeobox protein HHEX | Increased |
| **P80748** | IGLV3-21 | Immunoglobulin lambda variable 3-21 | Increased |
| **P02750** | LRG1 | Leucine-rich alpha-2-glycoprotein | Increased |
| **Q00994** | BEX3 | Protein BEX3 | Increased |
| **Q9H8W3** | FAM204A | Protein FAM204A | Increased |
| **O60248** | SOX15 | Protein SOX-15 | Increased |
| **P00734** | F2 | Prothrombin | Increased |
| **P0DJI8** | SAA1 | Serum amyloid A-1 protein | Increased |
| **P0DJI9** | SAA2 | Serum amyloid A-2 protein | Increased |
| **P05543** | SERPINA7 | Thyroxine-binding globulin | Increased |
| **Q86WR6** | C17orf64 | Uncharacterized protein C17orf64 | Increased |

**Table S2.** List of the 55 differentially expressed proteins identified in AML BM plasma from FAB-M0-M1 subtype patients compared with HD BM plasma samples.

| Accession Code | Symbol | Description | Expression |
|---|---|---|---|
| **P01834** | IGKC | Immunoglobulin kappa constant | Decreased |
| **P02749** | APOH | Beta-2-glycoprotein 1 | Decreased |
| **P0DOX7** | IGK | Immunoglobulin kappa light chain | Decreased |
| **A0M8Q6** | IGLC7 | Immunoglobulin lambda constant 7 | Decreased |
| **P60709** | ACTB | Actin_ cytoplasmic 1 | Decreased |
| **P63261** | ACTG1 | Actin_ cytoplasmic 2 | Decreased |
| **Q4LEZ3** | AARD | Alanine and arginine-rich domain-containing protein | Decreased |
| **P01008** | SERPINC1 | Antithrombin-III | Decreased |
| **P04114** | APOB | Apolipoprotein B-100 | Decreased |
| **P07360** | C8G | Complement component C8 gamma chain | Decreased |
| **P00751** | CFB | Complement factor B | Decreased |
| **P08603** | CFH | Complement factor H | Decreased |
| **Q6UXB4** | CLEC4G | C-type lectin domain family 4 member G | Decreased |
| **Q9Y600** | CSAD | Cysteine sulfinic acid decarboxylase | Decreased |
| **Q8WTR2** | DUSP19 | Dual specificity protein phosphatase 19 | Decreased |
| **P46926** | GNPDA1 | Glucosamine-6-phosphate isomerase 1 | Decreased |
| **P01764** | IGHV3-23 | Immunoglobulin heavy variable 3-23 | Decreased |
| **P01768** | IGHV3-30 | Immunoglobulin heavy variable 3-30 | Decreased |
| **P0DP02** | IGHV3-30-3 | Immunoglobulin heavy variable 3-30-3 | Decreased |
| **P0DP03** | IGHV3-30-5 | Immunoglobulin heavy variable 3-30-5 | Decreased |
| **P01772** | IGHV3-33 | Immunoglobulin heavy variable 3-33 | Decreased |
| **P01767** | IGHV3-53 | Immunoglobulin heavy variable 3-53 | Decreased |
| **A0A0C4DH42** | IGHV3-66 | Immunoglobulin heavy variable 3-66 | Decreased |
| **A0A0B4J1X5** | IGHV3-74 | Immunoglobulin heavy variable 3-74 | Decreased |
| **Q14624** | ITIH4 | Inter-alpha-trypsin inhibitor heavy chain H4 | Decreased |
| **P41227** | NAA10 | N-alpha-acetyltransferase 10 | Decreased |
| **P13796** | LCP1 | Plastin-2 | Decreased |
| **P62324** | BTG1 | Protein BTG1 | Decreased |
| **Q8N6L0** | CCDC155 | Protein KASH5 | Decreased |
| **Q9GZL8** | BPESC1 | Putative BPES syndrome breakpoint region protein | Decreased |
| **Q8N8H1** | ZNF321P | Putative protein ZNF321 | Decreased |
| **Q6ZTI0** | ZNF321P | Putative uncharacterized protein FLJ44636 | Decreased |
| **Q86VE3** | SATL1 | Spermidine/spermine N(1)-acetyltransferase-like protein 1 | Decreased |
| **P21453** | S1PR1 | Sphingosine 1-phosphate receptor 1 | Decreased |
| **Q86WV1** | SKAP1 | Src kinase-associated phosphoprotein 1 | Decreased |
| **A0A075B6N3** | TRBV24-1 | T cell receptor beta variable 24-1 | Decreased |
| **P0C1Z6** | TFPT | TCF3 fusion partner | Decreased |
| **Q9Y3Q3** | TMED3 | Transmembrane emp24 domain-containing protein 3 | Decreased |
| **Q2T9K0** | TMEM44 | Transmembrane protein 44 | Decreased |
| **P02766** | TTR | Transthyretin | Decreased |
| **Q03403** | TFF2 | Trefoil factor 2 | Decreased |
| **Q9BV90** | SNRNP25 | U11/U12 small nuclear ribonucleoprotein 25 kDa protein | Decreased |
| **P54284** | CACNB3 | Voltage-dependent L-type calcium channel subunit beta-3 | Decreased |
| **P01011** | SERPINA3 | Alpha-1-antichymotrypsin | Increased |
| **Q9H765** | ASB8 | Ankyrin repeat and SOCS box protein 8 | Increased |
| **P02656** | APOC3 | Apolipoprotein C-III | Increased |
| **P04196** | HRG | Histidine-rich glycoprotein | Increased |
| **A0A0B4J1U7** | IGHV6-1 | Immunoglobulin heavy variable 6-1 | Increased |
| **A0A075B6K0** | IGLV3-16 | Immunoglobulin lambda variable 3-16 | Increased |
| **P01717** | IGLV3-25 | Immunoglobulin lambda variable 3-25 | Increased |
| **P01718** | IGLV3-27 | Immunoglobulin lambda variable 3-27 | Increased |
| **Q02962** | PAX2 | Paired box protein Pax-2 | Increased |
| **Q8IXS6** | PALM2 | Paralemmin-2 | Increased |
| **Q9NWS6** | FAM118A | Protein FAM118A | Increased |
| **P0DJI8** | SAA1 | Serum amyloid A-1 protein | Increased |

**Table S3.** List of the 72 differentially expressed proteins identified in AML BM plasma from FAB-M3 subtype patients compared with HD BM plasma samples.

| Accession Code | Symbol | Description | Expression |
|---|---|---|---|
| **P0DOX2** | IGHA2 | Immunoglobulin alpha-2 heavy chain | Decreased |
| **P01876** | IGHA1 | Immunoglobulin heavy constant alpha 1 | Decreased |
| **P01877** | IGHA2 | Immunoglobulin heavy constant alpha 2 | Decreased |
| **P02749** | APOH | Beta-2-glycoprotein 1 | Decreased |
| **P60709** | ACTB | Actin_ cytoplasmic 1 | Decreased |
| **P63261** | ACTG1 | Actin_ cytoplasmic 2 | Decreased |
| **Q4LEZ3** | AARD | Alanine and arginine-rich domain-containing protein | Decreased |
| **P01008** | SERPINC1 | Antithrombin-III | Decreased |
| **P04114** | APOB | Apolipoprotein B-100 | Decreased |
| **P07360** | C8G | Complement component C8 gamma chain | Decreased |
| **P00751** | CFB | Complement factor B | Decreased |
| **P08603** | CFH | Complement factor H | Decreased |
| **Q6UXB4** | CLEC4G | C-type lectin domain family 4 member G | Decreased |
| **Q9Y600** | CSAD | Cysteine sulfinic acid decarboxylase | Decreased |
| **Q8WTR2** | DUSP19 | Dual specificity protein phosphatase 19 | Decreased |
| **P46926** | GNPDA1 | Glucosamine-6-phosphate isomerase 1 | Decreased |
| **P01762** | IGHV3-11 | Immunoglobulin heavy variable 3-11 | Decreased |
| **P01766** | IGHV3-13 | Immunoglobulin heavy variable 3-13 | Decreased |
| **A0A0C4DH32** | IGHV3-20 | Immunoglobulin heavy variable 3-20 | Decreased |
| **A0A0B4J1V1** | IGHV3-21 | Immunoglobulin heavy variable 3-21 | Decreased |
| **P01764** | IGHV3-23 | Immunoglobulin heavy variable 3-23 | Decreased |
| **P01768** | IGHV3-30 | Immunoglobulin heavy variable 3-30 | Decreased |
| **P0DP02** | IGHV3-30-3 | Immunoglobulin heavy variable 3-30-3 | Decreased |
| **P0DP03** | IGHV3-30-5 | Immunoglobulin heavy variable 3-30-5 | Decreased |
| **P01772** | IGHV3-33 | Immunoglobulin heavy variable 3-33 | Decreased |
| **A0A0B4J1X8** | IGHV3-43 | Immunoglobulin heavy variable 3-43 | Decreased |
| **P0DP04** | IGHV3-43D | Immunoglobulin heavy variable 3-43D | Decreased |
| **P01763** | IGHV3-48 | Immunoglobulin heavy variable 3-48 | Decreased |
| **P01767** | IGHV3-53 | Immunoglobulin heavy variable 3-53 | Decreased |
| **A0A0C4DH42** | IGHV3-66 | Immunoglobulin heavy variable 3-66 | Decreased |
| **P01780** | IGHV3-7 | Immunoglobulin heavy variable 3-7 | Decreased |
| **A0A0B4J1X5** | IGHV3-74 | Immunoglobulin heavy variable 3-74 | Decreased |
| **P01782** | IGHV3-9 | Immunoglobulin heavy variable 3-9 | Decreased |
| **P01624** | IGKV3-15 | Immunoglobulin kappa variable 3-15 | Decreased |
| **A0A087WSY6** | IGKV3D-15 | Immunoglobulin kappa variable 3D-15 | Decreased |
| **P41227** | NAA10 | N-alpha-acetyltransferase 10 | Decreased |
| **P00747** | PLG | Plasminogen | Decreased |
| **P13796** | LCP1 | Plastin-2 | Decreased |
| **P62324** | BTG1 | Protein BTG1 | Decreased |
| **Q8N6L0** | CCDC155 | Protein KASH5 | Decreased |
| **Q9GZL8** | BPESC1 | Putative BPES syndrome breakpoint region protein | Decreased |
| **Q8N8H1** | ZNF321P | Putative protein ZNF321 | Decreased |
| **Q6ZTI0** | YK032 | Putative uncharacterized protein FLJ44636 | Decreased |
| **Q86VE3** | SATL1 | Spermidine/spermine N(1)-acetyltransferase-like protein 1 | Decreased |
| **P21453** | S1PR1 | Sphingosine 1-phosphate receptor 1 | Decreased |
| **Q86WV1** | SKAP1 | Src kinase-associated phosphoprotein 1 | Decreased |
| **A0A075B6N3** | TRBV24 | T cell receptor beta variable 24-1 | Decreased |
| **P0C1Z6** | TFPT | TCF3 fusion partner | Decreased |
| **Q9Y3Q3** | TMED3 | Transmembrane emp24 domain-containing protein 3 | Decreased |

| | | | |
|---|---|---|---|
| **Q2T9K0** | TMEM44 | Transmembrane protein 44 | Decreased |
| **P02766** | TTR | Transthyretin | Decreased |
| **Q03403** | TFF2 | Trefoil factor 2 | Decreased |
| **Q9BV90** | SNRNP25 | U11/U12 small nuclear ribonucleoprotein 25 kDa protein | Decreased |
| **P54284** | CACNB3 | Voltage-dependent L-type calcium channel subunit beta-3 | Decreased |
| **Q8TDG2** | ACTRT1 | Actin-related protein T1 | Increased |
| **P01011** | SERPINA3 | Alpha-1-antichymotrypsin | Increased |
| **P02656** | APOC3 | Apolipoprotein C-III | Increased |
| **P10909** | CLU | Clusterin | Increased |
| **Q86T13** | CLEC14A | C-type lectin domain family 14 member A | Increased |
| **Q717R9** | CYS1 | Cystin-1 | Increased |
| **Q9UKR0** | KLK12 | Kallikrein-12 | Increased |
| **Q02962** | PAX2 | Paired box protein Pax-2 | Increased |
| **Q13522** | PPP1R1A | Protein phosphatase 1 regulatory subunit 1A | Increased |
| **P00734** | F2 | Prothrombin | Increased |
| **P02753** | RBP4 | Retinol-binding protein 4 | Increased |
| **Q9BQY4** | RHOXF2 | Rhox homeobox family member 2 | Increased |
| **P0C7M4** | RHOXF2B | Rhox homeobox family member 2B | Increased |
| **P0DJI8** | SAA1 | Serum amyloid A-1 protein | Increased |
| **P0DJI9** | SAA2 | Serum amyloid A-2 protein | Increased |
| **P05543** | SERPINA7 | Thyroxine-binding globulin | Increased |
| **Q9NPU4** | C14orf132 | Uncharacterized protein C14orf132 | Increased |
| **Q8N9V3** | WDSUB1 | WD repeat_ SAM and U-box domain-containing protein 1 | Increased |

**Table S4.** List of the 79 differentially expressed proteins identified in AML BM plasma from FAB-M4-M5 subtype patients compared with HD BM plasma samples.

| Accession Code | Symbol | Description | Expression |
|---|---|---|---|
| **P01877** | IGHA2 | Immunoglobulin heavy constant alpha 2 | Decreased |
| **P02100** | HBE1 | Hemoglobin subunit epsilon | Decreased |
| **P69891** | HBG1 | Hemoglobin subunit gamma-1 | Decreased |
| **P69892** | HBG2 | Hemoglobin subunit gamma-2 | Decreased |
| **P0DOX2** | IGA2 | Immunoglobulin alpha-2 heavy chain | Decreased |
| **P02042** | HBD | Hemoglobin subunit delta | Decreased |
| **P68871** | HBB | Hemoglobin subunit beta | Decreased |
| **P01876** | IGHA1 | Immunoglobulin heavy constant alpha 1 | Decreased |
| **P01859** | IGHG2 | Immunoglobulin heavy constant gamma 2 | Decreased |
| **P69905** | HBA1 | Hemoglobin subunit alpha | Decreased |
| **P60709** | ACTB | Actin_ cytoplasmic 1 | Decreased |
| **P63261** | ACTG1 | Actin_ cytoplasmic 2 | Decreased |
| **Q4LEZ3** | AARD | Alanine and arginine-rich domain-containing protein | Decreased |
| **P01008** | SERPINC1 | Antithrombin-III | Decreased |
| **P04114** | APOB | Apolipoprotein B-100 | Decreased |
| **P07360** | C8G | Complement component C8 gamma chain | Decreased |
| **P00751** | CFB | Complement factor B | Decreased |
| **P08603** | CFH | Complement factor H | Decreased |
| **Q6UXB4** | CLEC4G | C-type lectin domain family 4 member G | Decreased |
| **Q9Y600** | CSAD | Cysteine sulfinic acid decarboxylase | Decreased |
| **Q8WTR2** | DUSP19 | Dual specificity protein phosphatase 19 | Decreased |
| **P46926** | GNPDA1 | Glucosamine-6-phosphate isomerase 1 | Decreased |
| **P01762** | IGHV3-11 | Immunoglobulin heavy variable 3-11 | Decreased |
| **P01766** | IGHV3-13 | Immunoglobulin heavy variable 3-13 | Decreased |
| **A0A0C4DH32** | IGHV3-20 | Immunoglobulin heavy variable 3-20 | Decreased |
| **A0A0B4J1V1** | IGHV3-21 | Immunoglobulin heavy variable 3-21 | Decreased |
| **P01764** | IGHV3-23 | Immunoglobulin heavy variable 3-23 | Decreased |
| **P01768** | IGHV3-30 | Immunoglobulin heavy variable 3-30 | Decreased |
| **P0DP02** | IGHV3-30 | Immunoglobulin heavy variable 3-30-3 | Decreased |
| **P0DP03** | IGHV3-30-5 | Immunoglobulin heavy variable 3-30-5 | Decreased |
| **P01772** | IGHV3-33 | Immunoglobulin heavy variable 3-33 | Decreased |
| **A0A0B4J1X8** | IGHV3-43 | Immunoglobulin heavy variable 3-43 | Decreased |
| **P0DP04** | IGHV3-43D | Immunoglobulin heavy variable 3-43D | Decreased |
| **P01763** | IGHV3-48 | Immunoglobulin heavy variable 3-48 | Decreased |
| **P01767** | IGHV3-53 | Immunoglobulin heavy variable 3-53 | Decreased |
| **A0A0C4DH42** | IGHV3-66 | Immunoglobulin heavy variable 3-66 | Decreased |
| **P01780** | IGHV3-7 | Immunoglobulin heavy variable 3-7 | Decreased |
| **A0A0B4J1X5** | IGHV3-74 | Immunoglobulin heavy variable 3-74 | Decreased |
| **P01782** | IGHV3-9 | Immunoglobulin heavy variable 3-9 | Decreased |
| **P01624** | IGKV3-15 | Immunoglobulin kappa variable 3-15 | Decreased |
| **A0A087WSY6** | IGKV3D-15 | Immunoglobulin kappa variable 3D-15 | Decreased |
| **P41227** | NAA10 | N-alpha-acetyltransferase 10 | Decreased |
| **P13796** | LCP1 | Plastin-2 | Decreased |
| **P62324** | BTG1 | Protein BTG1 | Decreased |
| **Q8N6L0** | CCDC155 | Protein KASH5 | Decreased |
| **Q9GZL8** | BPESC1 | Putative BPES syndrome breakpoint region protein | Decreased |
| **Q6ZTI0** | YK032 | Putative uncharacterized protein FLJ44636 | Decreased |
| **Q86VE3** | SATL1 | Spermidine/spermine N(1)-acetyltransferase-like protein 1 | Decreased |
| **P21453** | S1PR1 | Sphingosine 1-phosphate receptor 1 | Decreased |

| | | | |
|---|---|---|---|
| **Q86WV1** | SKAP1 | Src kinase-associated phosphoprotein 1 | Decreased |
| **A0A075B6N3** | TRBV24-1 | T cell receptor beta variable 24-1 | Decreased |
| **P0C1Z6** | TFPT | TCF3 fusion partner | Decreased |
| **Q9Y3Q3** | TMED3 | Transmembrane emp24 domain-containing protein 3 | Decreased |
| **Q2T9K0** | TMEM44 | Transmembrane protein 44 | Decreased |
| **Q03403** | TFF2 | Trefoil factor 2 | Decreased |
| **Q9BV90** | SNRNP25 | U11/U12 small nuclear ribonucleoprotein 25 kDa protein | Decreased |
| **P54284** | CACNB3 | Voltage-dependent L-type calcium channel subunit beta-3 | Decreased |
| **Q96SZ5** | ADO | 2-aminoethanethiol dioxygenase | Increased |
| **P14550** | AKR1A1 | Aldo-keto reductase family 1 member A1 | Increased |
| **P01011** | SERPINA3 | Alpha-1-antichymotrypsin | Increased |
| **Q96DE5** | ANAPC16 | Anaphase-promoting complex subunit 16 | Increased |
| **Q9H765** | ASB8 | Ankyrin repeat and SOCS box protein 8 | Increased |
| **P02656** | APOC3 | Apolipoprotein C-III | Increased |
| **P02649** | APOE | Apolipoprotein E | Increased |
| **Q8WUZ0** | BCL7C | B-cell CLL/lymphoma 7 protein family member C | Increased |
| **Q30KQ8** | DEFB112 | Beta-defensin 112 | Increased |
| **Q9Y2B9** | PKIG | cAMP-dependent protein kinase inhibitor gamma | Increased |
| **Q96L91** | EP400 | E1A-binding protein p400 | Increased |
| **P06396** | GSN | Gelsolin | Increased |
| **P19827** | ITIH1 | Inter-alpha-trypsin inhibitor heavy chain H1 | Increased |
| **P16860** | NPPB | Natriuretic peptides B | Increased |
| **Q9UJ90** | KCNE5 | Potassium voltage-gated channel subfamily E regulatory beta subunit 5 | Increased |
| **P00734** | F2 | Prothrombin | Increased |
| **Q6ZTU2** | EP400P1 | Putative EP400-like protein | Increased |
| **Q9NVA2** | SEPT11 | Septin-11 | Increased |
| **P0DJI8** | SAA1 | Serum amyloid A-1 protein | Increased |
| **Q8NBI5** | SLC43A3 | Solute carrier family 43 member 3 | Increased |
| **Q9NZC2** | TREM2 | Triggering receptor expressed on myeloid cells 2 | Increased |
| **Q9BQY6** | WFDC6 | WAP four-disulfide core domain protein 6 | Increased |

**Disclaimer/Publisher's Note:** The statements, opinions and data contained in all publications are solely those of the individual author(s) and contributor(s) and not of MDPI and/or the editor(s). MDPI and/or the editor(s) disclaim responsibility for any injury to people or property resulting from any ideas, methods, instructions or products referred to in the content.
